# Supplementary material for: Inter-species gene flow drives ongoing evolution of Streptococcus pyogenes and Streptococcus dysgalactiae subsp. equisimilis
Source: Nat Commun. 2024 Mar 13;15:2286. doi: 10.1038/s41467-024-46530-2 (PMC10937727; doi:10.1038/s41467-024-46530-2)
Supplement: Supplementary file 3 — Description of Additional Supplementary Files [file 41467_2024_46530_MOESM3_ESM.pdf]

Description of Additional Supplementary Files

Supplementary Data 1. a) *Streptococcus dysgalactiae* subsp. *equisimilis* isolates included in this study with associated metadata. **b)** Virulence factor, antimicrobial resistance, and putative regulator screen. Putative stand-alone and two-component regulators were defined as by Buckley *et al.* [1, 2]

Supplementary Data 2. a) List of 837 *Streptococcus dysgalactiae* subsp. *equisimilis* core genes identified by fastGEAR as having recombinogenic signatures. fastGEAR infers "recent" events as affecting individual isolates in the dataset whereas "ancestral" events affect an entire cluster of sequences. GGS_124 RefSeq locus tags (NC_012891.1) are given where available. Where not available, NC_019042.1 or NC_018712.1 locus tags are given except for 2 genes which were missing in all three RefSeq reference genomes. A small number of genes were merged in the pangenome by Panaroo[3] and locus tags are delimited by ";". A small number of genes were "refound" where additional annotations are inferred by Panaroo through comparison across the pangenome. **b)** List of 1,116 genes core in both *Streptococcus dysgalactiae* subsp. *equisimilis* (SDSE) and *S. pyogenes* analysed for putative cross-species recombination events by fastGEAR[4]. Core genes present in >99% of SDSE and *S. pyogenes* isolates with less than 25% length variation were included in the analysis. Of 1,166 core genes, 526 had at least two sequence clusters inferred by fastGEAR and at least one of which had members from both species (shared clusters). These may reflect whole gene recombination or shared ancestry. Furthermore, 393 genes with unique reference genome GGS_124 (NC_012891.1) SDSE locus tags had predicted recombination from a single-species sequence cluster to a genome from the other species (cross species events). For genes where a GGS_124

locus tag was not available, the locus tag from SDSE reference genome AC-2713 (NC_019042.1) was given.

Supplementary Data 3. a) Prophage, integrative conjugative element and mobility element *Streptococcus dysgalactiae* subsp. *equisimilis* insertion regions mapped to the GGS_124 reference genome (NC_012891.1). **b)** Alternative *Streptococcus dysgalactiae* subsp. *equisimilis* insertion regions with fully assembled elements reflective of likely genome rearrangements around mobile genetic elements. Insertion regions were mapped to the GGS_124 reference genome (NC_012891.1). **c)** *Streptococcus pyogenes* integrative conjugative element and prophage insertion regions mapped to the *S. pyogenes* reference genome SF370 (NC_002737.2). Homologous *Streptococcus dysgalactiae* subsp. *equisimilis* (SDSE) insertion region no. refers to insertion region numbers given in Fig. 3a.

Supplementary Data 4. List of 665 'non-recombinogenic' *Streptococcus dysgalactiae* subsp. *equisimilis* core genes and 376 'non-recombinogenic' *Streptococcus pyogenes* core genes with markers of selection pressure (ratio of non-synonymous d_N and synonymous d_S codon substitutions). d_N/d_S was calculated alignment-wide for each gene and by codon/site. Some shared core genes had d_N/d_S calculated for only a single species due to inferred intraspecies recombination affecting only one species.

Supplementary references

1. Buckley SJ, Davies MR, McMillan DJ. In silico characterisation of stand-alone response regulators of Streptococcus pyogenes. *PLoS One* 2020; **15**: e0240834.

2. Buckley SJ, Timms P, Davies MR et al. In silico characterisation of the two-component system regulators of Streptococcus pyogenes. *PLoS One* 2018; **13**: e0199163.

3. Tonkin-Hill G, MacAlasdair N, Ruis C et al. Producing polished prokaryotic pangenomes with the Panaroo pipeline. *Genome Biol* 2020; **21**: 180.

4. Mostowy R, Croucher NJ, Andam CP et al. Efficient Inference of Recent and Ancestral Recombination within Bacterial Populations. *Mol Biol Evol* 2017; **34**: 1167-82.
