# Supplementary material for: Inter-species gene flow drives ongoing evolution of Streptococcus pyogenes and Streptococcus dysgalactiae subsp. equisimilis
Source: Nat Commun. 2024 Mar 13;15:2286. doi: 10.1038/s41467-024-46530-2 (PMC10937727; doi:10.1038/s41467-024-46530-2)
Supplement: Supplementary file 8 — Reporting Summary [file 41467_2024_46530_MOESM8_ESM.pdf]

nature portfolio

Corresponding author(s): Dr Mark Davies

Last updated by author(s): Sep 16, 2024

Reporting Summary

Nature Portfolio wishes to improve the reproducibility of the work that we publish. This form provides structure for consistency and transparency in reporting. For further information on Nature Portfolio policies, see our Editorial Policies and the Editorial Policy Checklist.

Statistics

For all statistical analyses, confirm that the following items are present in the figure legend, table legend, main text, or Methods section.

n/a	Confirmed	
☐	☒	The exact sample size (*n*) for each experimental group/condition, given as a discrete number and unit of measurement
☒	☐	A statement on whether measurements were taken from distinct samples or whether the same sample was measured repeatedly
☐	☒	The statistical test(s) used AND whether they are one- or two-sided<br>*Only common tests should be described solely by name; describe more complex techniques in the Methods section.*
☐	☒	A description of all covariates tested
☐	☒	A description of any assumptions or corrections, such as tests of normality and adjustment for multiple comparisons
☐	☒	A full description of the statistical parameters including central tendency (e.g. means) or other basic estimates (e.g. regression coefficient) AND variation (e.g. standard deviation) or associated estimates of uncertainty (e.g. confidence intervals)
☐	☒	For null hypothesis testing, the test statistic (e.g. *F*, *t*, *r*) with confidence intervals, effect sizes, degrees of freedom and *P* value noted<br>*Give P values as exact values whenever suitable.*
☐	☒	For Bayesian analysis, information on the choice of priors and Markov chain Monte Carlo settings
☒	☐	For hierarchical and complex designs, identification of the appropriate level for tests and full reporting of outcomes
☒	☐	Estimates of effect sizes (e.g. Cohen's *d*, Pearson's *r*), indicating how they were calculated

Our web collection on statistics for biologists contains articles on many of the points above.

Software and code

Policy information about availability of computer code

Data collection	No software was used for data collection
Data analysis	All custom code and scripts used to analyse the data are publicly available at https://github.com/OuliXie/Global_SDSE.<br>Software packages used in the analysis include Kraken2 v2.1.2, Shovill v1.1.0 with SPAdes assembler v3.14.0, Prokka v1.14.6, emmtyper v0.2.0, MLST v2.22.0, SMRT analysis system v2.3.0.140936, Quiver v1, BridgeMapper v1, socru v2.2.4, Verticall v0.4.0, fastME v2.1.6.1, Snippy v4.6.0, IQ-tree v2.0.6, TreeDist v2.5.0, BactDating v1.1, Gubbins v3.1.2, PopPUNK v2.4.0, PHYLOViZ v2.0, screen_assembly v1.2.7, Abricate v1.0.1, Magphi v1.0.1, Abritamr v1.0.6 with AMRfinder plus v3.10.18, Panaroo v1.2.10, eggNOG-mapper v2.1.7, Corekaburra v0.0.2, HMMER v3.3.2, fastGEAR, MAFFT v7.505, mge-cluster v1.0.2, Easyfig v2.2.3, CD-HIT v4.8.1, and HyPhy v2.5.58.

For manuscripts utilizing custom algorithms or software that are central to the research but not yet described in published literature, software must be made available to editors and reviewers. We strongly encourage code deposition in a community repository (e.g. GitHub). See the Nature Portfolio guidelines for submitting code & software for further information.

Data

Policy information about availability of data

All manuscripts must include a data availability statement. This statement should provide the following information, where applicable:

- Accession codes, unique identifiers, or web links for publicly available datasets
- A description of any restrictions on data availability
- For clinical datasets or third party data, please ensure that the statement adheres to our policy

Reads for newly sequenced genomes have been deposited in the European Nucleotide Archive (https://www.ebi.ac.uk/ena/). Accessions for raw sequencing data are available in Supplementary Table 1a. The complete genome sequence of S. dysgalactiae subsp. equisimilis NS3396 was deposited to GenBank (accession CP128987, https://www.ncbi.nlm.nih.gov/genbank/). Source data are provided with this paper. The authors confirm all supporting data have been provided within the article or in supplementary data files.

Research involving human participants, their data, or biological material

Policy information about studies with human participants or human data. See also policy information about sex, gender (identity/presentation), and sexual orientation and race, ethnicity and racism.

Reporting on sex and gender	N/A
Reporting on race, ethnicity, or other socially relevant groupings	N/A
Population characteristics	N/A
Recruitment	N/A
Ethics oversight	N/A

Note that full information on the approval of the study protocol must also be provided in the manuscript.

Field-specific reporting

Please select the one below that is the best fit for your research. If you are not sure, read the appropriate sections before making your selection.

☒ Life sciences ☐ Behavioural & social sciences ☐ Ecological, evolutionary & environmental sciences

For a reference copy of the document with all sections, see nature.com/documents/nr-reporting-summary-flat.pdf

Life sciences study design

All studies must disclose on these points even when the disclosure is negative.

Sample size	No formal sample size calculations were performed. However, where available, samples were chosen to be as broadly representative from regions around the globe as possible - a total of 228 genomes are newly reported with this study. All publicly available Streptococcus dysgalactiae subsp. equisimilis genomes available as of 4 May 2022 were screened and included to maximise diversity - 273 publicly available genomes were included after quality control and screened to minimise over-representation of closely related clones. A dataset of 2,083 globally disseminated S. pyogenes genomes previously published by Davies et al. was included which was again chosen to maximise global diversity of strains.
Data exclusions	Genomes which failed quality control checks as described in the methods were excluded from the analysis.
Replication	Seeds (123) were used for Bayesian dating of phylogenetic trees to ensure reproducibility. Custom code to classify mobile genetic elements from the pangenome is publicly available at https://github.com/OuliXie/Global_SDSE.
Randomization	No randomisation was performed. Isolates were allocated to their respective species (Streptococcus dysgalactiae subsp. equisimilis or S. pyogenes) based on kraken2 and MLST classification for cross-species comparisons.
Blinding	No blinding was performed in this study. Blinding was not required as the results are quantitative and did not require subjective judgment or interpretation.

Reporting for specific materials, systems and methods

We require information from authors about some types of materials, experimental systems and methods used in many studies. Here, indicate whether each material, system or method listed is relevant to your study. If you are not sure if a list item applies to your research, read the appropriate section before selecting a response.

Materials & experimental systems

n/a	Involved in the study
☒	☐ Antibodies
☒	☐ Eukaryotic cell lines
☒	☐ Palaeontology and archaeology
☒	☐ Animals and other organisms
☒	☐ Clinical data
☒	☐ Dual use research of concern
☒	☐ Plants

Methods

n/a	Involved in the study
☒	☐ ChIP-seq
☒	☐ Flow cytometry
☒	☐ MRI-based neuroimaging

Plants

Seed stocks

Report on the source of all seed stocks or other plant material used. If applicable, state the seed stock centre and catalogue number. If plant specimens were collected from the field, describe the collection location, date and sampling procedures.

Novel plant genotypes

Describe the methods by which all novel plant genotypes were produced. This includes those generated by transgenic approaches, gene editing, chemical/radiation-based mutagenesis and hybridization. For transgenic lines, describe the transformation method, the number of independent lines analyzed and the generation upon which experiments were performed. For gene-edited lines, describe the editor used, the endogenous sequence targeted for editing, the targeting guide RNA sequence (if applicable) and how the editor was applied.

Authentication

Describe any authentication procedures for each seed stock used or novel genotype generated. Describe any experiments used to assess the effect of a mutation and, where applicable, how potential secondary effects (e.g. second site T-DNA insertions, mosiacism, off-target gene editing) were examined.
